# Surgical outcomes of laparoscopic distal gastrectomy compared to open distal gastrectomy: A retrospective cohort study based on a nationwide registry database in Japan

**DOI:** 10.1002/ags3.12054

**Published:** 2017-12-22

**Authors:** Kazuhiro Yoshida, Michitaka Honda, Hiraku Kumamaru, Yasuhiro Kodera, Yoshihiro Kakeji, Naoki Hiki, Tsuyoshi Etoh, Hiroaki Miyata, Yuichi Yamashita, Yasuyuki Seto, Seigo Kitano, Hiroyuki Konno

**Affiliations:** ^1^ Graduate School of Medicine Department of Surgical Oncology Gifu University Gifu Japan; ^2^ Department of Minimally Invasive Medical and Surgical Oncology Fukushima Medical University Fukushima Japan; ^3^ Department of Healthcare Quality Assessment Graduate School of Medicine University of Tokyo Tokyo Japan; ^4^ Department of Gastroenterological Surgery Graduate School of Medicine Nagoya University Nagoya Japan; ^5^ Division of Gastrointestinal Surgery Department of Surgery Graduate School of Medicine Kobe University Kobe Japan; ^6^ Department of Gastroenterological Surgery Gastroenterological Center Cancer Institute Hospital of Japanese Foundation for Cancer Research Tokyo Japan; ^7^ Faculty of Medicine Department of Gastroenterological and Pediatric Surgery Oita University Oita Japan; ^8^ Department of Health Policy and Management School of Medicine Keio University Tokyo Japan; ^9^ Department of Gastroenterological Surgery Fukuoka University Faculty of Medicine Fukuoka Japan; ^10^ The Japanese Society of Gastroenterological Surgery Tokyo Japan; ^11^ Oita University Oita Japan; ^12^ Hamamatsu University School of Medicine Hamamatsu Japan

**Keywords:** gastric cancer, laparoscopic surgery, national clinical database, open gastrectomy, propensity score matching

## Abstract

To clarify the safety profile of laparoscopic distal gastrectomy (LDG) for gastric cancer patients, the short‐term outcome of LDG was compared to that of open distal gastrectomy (ODG) by propensity score matching using data from the Japanese National Clinical Database (NCD). We conducted a retrospective cohort study of patients undergoing distal gastrectomy between January 2012 and December 2013. Using the data for 70 346 patients registered in the NCD, incidences of mortality and morbidities were compared between LDG patients and ODG patients in the propensity score matched stage I patients (ODG: n = 14 386, LDG: n = 14 386) and stage II‐IV patients (ODG: n = 3738, LDG: n = 3738), respectively. There was no significant difference in mortality rates between LDG and ODG at all stages. Operating time was significantly longer in LDG compared to ODG, whereas blood loss and incidences of superficial surgical site infection (SSI), deep SSI, and wound dehiscence were significantly higher in ODG at all stages. Interestingly, pancreatic fistula was found significantly more often in LDG (1%) compared to ODG (0.8%) (*P* = .01) in stage I patients; however, it was not different in stage II‐IV patients. The length of postoperative stay was significantly longer in patients undergoing ODG compared to LDG at all stages. LDG in general practice might be a feasible therapeutic option in patients with both advanced gastric cancer and those with early gastric cancer in Japan.

## 1 INTRODUCTION

The incidence of gastric cancer (GC) is decreasing, but it still remains the second leading cause of death worldwide.^1^ In Japan, according to the national cancer registry, 40%‐50% of GC patients are detected at an early stage, and they are mostly treated by minimally invasive surgery including endoscopic mucosal resection and laparoscopic surgery.

Laparoscopic distal gastrectomy (LDG) was initially reported in 1991^2^ and has recently become prevalent. Its short‐term outcomes have been clarified by randomized controlled trials (RCT), and operative procedures have been recommended at level B in the Japanese GC cancer treatment guidelines.^3^ However, the results of RCT may not necessarily represent the effectiveness of the procedure in general practice; the patients enrolled in the clinical trials are mostly in good condition as trials tend to have eligibility criteria that prohibit the enrollment of high‐risk patients such as the elderly and patients with severe comorbidities. Moreover, the hospitals participating in the RCT are mostly high‐volume centers, and the qualities of treatment and care do not necessarily represent those of the routine care provided at community hospitals.^4^, ^5^, ^6^, ^7^ This is especially true in the field of laparoscopic surgery, which requires special training to acquire proficiency in high‐quality techniques.^8^ It is obvious to postulate that there should be differences in treatment outcomes between high‐volume centers and hospitals in general and between doctors with and without board certification by special academic societies. In the past decade, LDG has been carried out in general practice, and the number carried out in 2013 increased 6‐fold compared to that of 2001, without any evaluation or quality control that would be warranted from the concerns described above.^9^ It is only recently that we have begun to pay close attention to the quality and outcomes of these procedures, which are being conducted at institutions all over Japan.

To solve the problems mentioned above, analysis using large, truly trustable, and real‐time data is necessary. The Japanese National Clinical Database (NCD) is a nationwide web‐based data entry system started in 2011 that is based on the National Surgical Quality Improvement Program of the American College of Surgeons.^10^ The NCD is the largest clinical database in Japan, covering more than 90% of the general clinical practice data relating to surgery and surveying the operative risks and complications of approximately 1.2 million cases from 4105 institutions per year. It was founded in April 2010 by the Japan Surgical Society and other societies. According to the annual report of the NCD in 2013, the total number of 115 nominated gastroenterological operations carried out from January 2011 to December 2012 was 949 824. The procedures were done on the esophagus (1.7%), gastroduodenal area (15.0%), small intestine and colon (35.4%), anorectal area (9.6%), liver (5.2%), gall bladder (23.8%), pancreas (3.1%), and others (5.5%). Now, the NCD data are regarded as the most reliable baseline data reflecting general practice. The enrolled cases are linked with the board certification system for surgeons, and enrollment is also mandatory for teaching hospitals.

The present study, the largest cohort study to date, was conducted to clarify the present situation of LDG in general practice and also to confirm that LDG is conducted safely in patients with advanced GC and in those with early GC in Japan.

## 2 MATERIALS AND METHODS

### 2.1 Study design and cohort development

This study was a retrospective cohort study enrolling patients registered in the NCD gastrointestinal surgery registry as undergoing distal gastrectomy during the enrollment period between January 2012 and December 2013 and the study was conducted as a collaborative study with Japanese Gastric Cancer Association, Japan Society for Endoscopic Surgery, the Japanese Society of Gastroenterological Surgery and NCD. We divided the cohort into patients with stage I and those with stages II‐IV GC and analyzed them separately as treatments between these two groups are distinct. All procedures were conducted in accordance with the ethical standards of the respective committees on human experimentation (institutional and national) and with the Helsinki Declaration of 1964 and later versions. The study was approved by the Institutional Review Board of Gifu University.

### 2.2 Outcomes and identification of confounding factors

Because many confounding factors were expected when comparing conventional open distal gastrectomy (ODG) against LDG for the incidences of perioperative events, we held a consensus meeting that included members such as laparoscopy surgeons, gastroenterology surgeons, and clinical epidemiologists to determine the study outcomes and the confounding variables. We defined the primary endpoints as 30‐day postoperative death and surgical death (deaths within 30 days after surgery or those occurring while hospitalized, respectively). Secondary endpoints included occurrences of reoperation, readmission, and operative complications, and operating time, blood loss, and length of postoperative stay. Confounding factors included patients’ age, gender, American Society of Anesthesiologists performance status (ASA‐PS) score, and body mass index (BMI); preoperative conditions including weight loss of greater than 10% within the past 6 months, smoking status, emergency ambulance to the hospital, presence of habitual alcohol intake, and patient activities of daily living; presence of comorbidities including insulin‐dependent diabetes mellitus, respiratory disease, chronic obstructive pulmonary disease, hypertension, angina, hemodialysis, congestive heart failure, history of cerebrovascular accident, long‐term use of steroids, and bleeding disorder; surgical tumor node metastasis (TNM) classifications, presence of concurrent cholecystectomy, whether the surgery was emergent, and the presence of preoperative chemotherapy. We also considered as potential confounders of the present study laboratory abnormalities that were identified as being strongly associated with perioperative mortality in past studies.

### 2.3 Propensity score matching and statistical analysis

A statistical analyst conducted propensity score modeling and matching while being blinded to the outcome. The propensity score was estimated by logistic regression models predicting the exposure of undergoing laparoscopic surgery against undergoing ODG from the above‐described confounding variables, but built separately in the two cohorts for stage IA + IB cases and stage II‐IV cases. After propensity score estimation, each patient undergoing laparoscopy was matched to a patient undergoing open surgery using the macro, a SAS software program made public, by Marcelo Coca‐Perraillon,^11^ through a greedy matching algorithm without replacement with a matching caliper of 0.2 standard deviation of logit of the propensity score. We assessed the balance of the matched cohort by calculating the standardized difference between the two groups using the macro devised by Yang and Dalton.^12^ We estimated the occurrences of primary and secondary outcomes in the matched cohort and compared them between the two surgical approach groups using Fisher's exact test for the outcomes with an expected cell count less than 5 or Pearson's chi‐squared test for others for binary variables and the Wilcoxon rank‐sum test for continuous variables. Comparisons were all two‐sided, and *P*‐values less than .05 were considered significant. All analyses were conducted using SAS 9.4 statistical software (SAS Institute, Cary, NC, USA).

## 3 RESULTS

### 3.1 Patient characteristics

**Figure 1:**
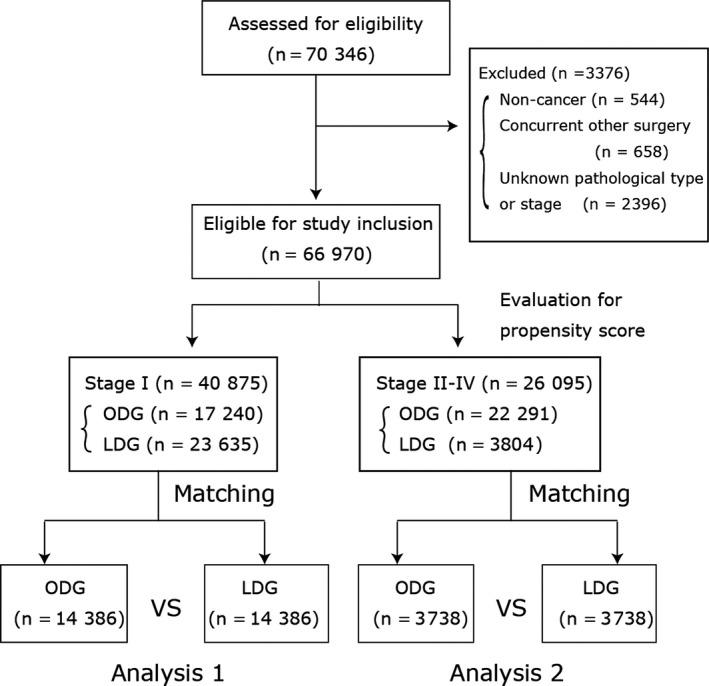
Flowchart of the participants enrolled as undergoing distal gastrectomy in the National Clinical Database. LDG, laparoscopic distal gastrectomy; ODG, open distal gastrectomy

We initially enrolled 70 346 patients in the NCD database who underwent distal gastrectomy during the study period. After excluding cases of non‐cancers (n = 544), cases with concurrent surgical procedures other than cholecystectomy (n = 658), and those of surgical stage 0 or undefined (n = 2396), we were left with 40 875 patients with stage IA or IB cancer and 26 095 patients with stage II‐IV cancer. A flowchart of the enrollment and exclusion of cases is depicted in Figure 1.

Background characteristics of the surgical stage I patients are shown in Table 1. Information on pre‐matching of patients is tabulated on the left side, whereas that of the propensity‐matched patients is tabulated on the right. Among the total of 40 875 patients, 17 240 underwent open surgery and 23 635 underwent laparoscopic surgery. Patients undergoing open surgery were more likely to be older, have poorer ASA‐PS, and have more comorbidities, including insulin‐dependent diabetes mellitus and respiratory disease, compared to those undergoing laparoscopic surgery. Moreover, the ODG patients comprised a higher percentage of stage IB patients than the LDG patients, suggesting that higher‐risk patients tended to undergo open surgery. After propensity score matching, these discrepancies in patient background disappeared (standardized difference 0.05 or less) (open surgery: n = 14 386, laparoscopic surgery: n = 14 386) as shown in Table 1.

**Table 1:** Baseline characteristics before and after propensity score matching in stage IA/IB patients

	All patients (n = 40 875)					Propensity‐matched patients (n = 28 772)				
	ODG (n = 17 240)	%	LDG (n = 23 635)	%	Standardized difference	ODG (n = 14 386)	%	LDG (n = 14 386)	%	Standardized difference
Age (y)										
Median [IQR]	71 [63‐78]		68 [61‐75]		−0.27	69.1 (11.0)		68.5 (11.2)		−0.05
<65	4981	28.9	9060	38.3	0.25	4536	31.5	4563	31.7	0.01
65‐75	5786	33.6	8186	34.6		4970	34.5	4988	34.7	
75<	6473	37.5	6389	27.0		4880	33.9	4835	33.6	
Gender										
Male	11 794	68.4	15 424	65.3	0.07	9770	67.9	9794	68.1	0.00
Female	5446	31.6	8211	34.7		4616	32.1	4592	31.9	
ASA‐PS										
≤2	15 373	89.2	22 231	94.1	−0.18	13 202	91.8	13 189	91.7	0.00
≥3	1867	10.8	1404	5.9		1184	8.2	1197	8.3	
BMI (kg/m^2^)										
Mean (SD)	22.6 (3.4)		22.6 (3.2)		0.00	22.7 (3.4)		22.7 (3.3)		0.00
Weight loss 10%<	366	2.1	284	1.2	−0.07	238	1.7	232	1.6	0.00
Smoking	3387	19.6	4982	21.1	0.04	2941	20.4	2926	20.3	0.00
Habitual alcohol intake	4519	26.2	7107	30.1	0.09	4013	27.9	4010	27.9	0.00
Comorbidities										
Diabetes mellitus	562	3.3	560	2.4	−0.05	413	2.9	402	2.8	0.00
Respiratory	320	1.9	235	1.0	−0.07	197	1.4	201	1.4	0.00
COPD	648	3.8	786	3.3	−0.02	524	3.6	522	3.6	0.00
Hypertension	6779	39.3	8147	34.5	−0.10	5496	38.2	5480	38.1	0.00
Ischemic heart disease	270	1.6	264	1.1	−0.04	204	1.4	194	1.3	−0.01
Dialysis	241	1.4	137	0.6	−0.08	129	0.9	125	0.9	0.00
Cerebrovascular event	348	2.0	254	1.1	−0.08	219	1.5	217	1.5	0.00
Use of steroid	155	0.9	180	0.8	−0.02	117	0.8	125	0.9	0.01
Bleeding disorder	701	4.1	593	2.5	−0.09	483	3.4	485	3.4	0.00
Surgical T										
1a	5256	30.5	9263	39.2	0.37	4846	33.7	4916	34.2	0.01
1b	8307	48.2	12 340	52.2		7480	52.0	7458	51.8	
2	3677	21.3	2032	8.6		2060	14.3	2012	14.0	
Surgical N										
N0	16 073	93.2	22 557	95.4	0.18	13 498	93.8	13 482	93.7	0.01
N1	1167	6.8	1078	4.6		888	6.2	904	6.3	
Surgical stage										
IA	12 396	71.9	20 525	86.8	0.18	11 438	79.5	11 470	79.7	0.01
IB	4844	28.1	3110	13.2		2948	20.5	2916	20.3	
Preoperative chemotherapy	139	0.8	98	0.4	−0.05	83	0.6	87	0.6	0.00
Cholecystectomy	2709	15.7	1799	7.6	−0.25	1774	12.3	1732	12.0	−0.01
Emergency surgery	124	0.7	68	0.3	−0.06	53	0.4	64	0.4	0.01

ASA‐PS, American Society of Anesthesiologists physical status; BMI, body mass index; COPD, chronic obstructive pulmonary disease; IQR, interquartile range; LDG, laparoscopic distal gastrectomy; ODG, open distal gastrectomy; SD, standard deviation.

**Figure 2:**
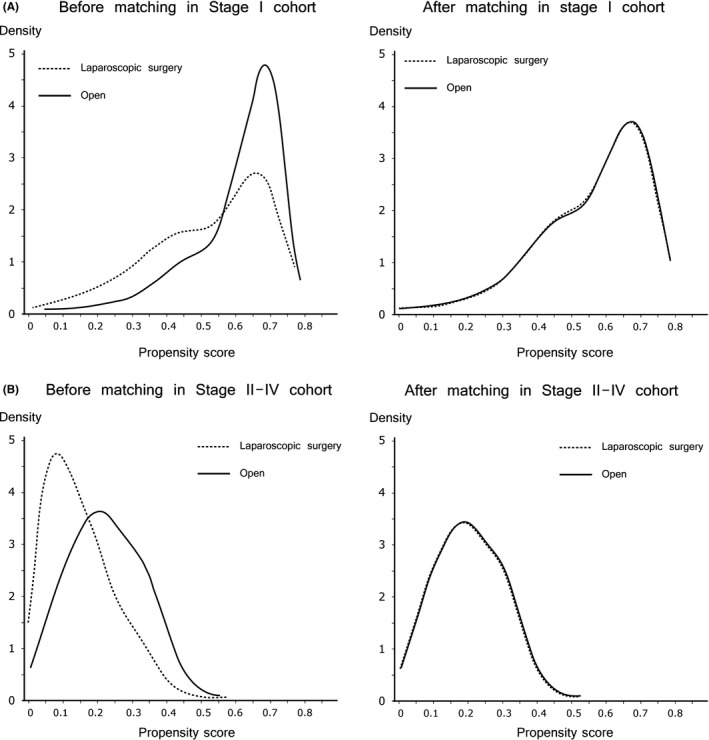
Propensity score distribution in the whole cohort and in the matched cohort

Characteristics of patients with surgical stage II‐IV cancers (all patients: n = 26 095, ODG: n = 22 291, LDG: n = 3804) are shown in Table 2. Open surgeries were more likely to be conducted on patients with poorer ASA‐PS, with higher frequency of bodyweight loss above 10%, poorer activities of daily living, and more respiratory disease. The percentage of patients with stage II cancer was much higher in the LDG group than in the ODG group. Surprisingly, 5.8% of patients undergoing laparoscopic surgery had stage IV GC. After propensity score matching, we had 3738 patients each undergoing LDG and ODG, with the standardized difference at 0.05 or less for all of the above characteristics. Figure 2 depicts the distribution of the propensity score in the whole cohort and in the matched cohort.

**Table 2:** Surgical outcomes in Stage IA/IB patients

	ODG (n = 14 386)	(%)	LDG (n = 14 386)	(%)	*P*‐value
Operating time (min)					
Median [percentail 10‐90]	209 [130‐315]		287 [194‐406]		<.001
Blood loss (mL)					
Median [percentail 10‐90]	185 [50‐547]		50 [1‐250]		<.001
Mortality					
Within 30 days	33	0.23	31	0.22	.90
In‐hospital	76	0.53	55	0.38	.08
Readmission within 30 days	263	1.83	292	2.03	.23
Reoperation	290	2.02	317	2.20	.29
Complications					
Superficial SSI	266	1.85	149	1.04	<.001
Deep SSI	89	0.62	55	0.38	<.01
Intra‐abdominal abscess	289	2.01	281	1.95	.76
Leakage	257	1.79	274	1.90	.48
Pancreatic fistula (grade B,C)	116	0.81	145	1.01	.01
Wound dehiscence	59	0.41	27	0.19	<.001
Pneumonia	240	1.67	195	1.36	.03
Pulmonary embolism	15	0.10	11	0.08	.56
Sepsis	44	0.31	37	0.26	.50
Length of postoperative stay					
Median [percentail 10‐90]	15 [10‐31]		12 [8‐24]		<.001

*P*‐values derived from Wilcoxon rank‐sum test for continuous variables and Pearson's chi‐squared or Fisher's exact test for binary variables.

LDG, laparoscopic distal gastrectomy; ODG, open distal gastrectomy; SSI, surgical site infection.

### 3.2 Mortality and complications in stage I GC patients

Mortality and complications for propensity‐matched patients with stage I GC (n = 28 772) are shown in Table 3. We observed no significant difference in either 30‐day or in‐hospital mortality: 33 (0.2%) and 31 (0.2%) deaths within 30 days after ODG and LDG (*P* = .90) and 76 (0.5%) and 55 (0.4%) patients with in‐hospital mortality after ODG and LDG (*P* = .08), respectively. In addition, there were no significant differences in the incidences of reoperation and readmission after surgery. Mean and median operating times were significantly longer in LDG (mean: 295 minutes, median: 287 minutes) compared to ODG (mean: 218 minutes, median: 209 minutes), whereas blood loss was significantly higher in ODG (mean: 264.5 mL, median: 185 mL) compared to LDG (mean: 105.8 mL, median: 50 mL). Length of postoperative stay was significantly longer in ODG (median: 15 days, 10th‐90th percentile: 10‐31 days) compared to LDG (median: 12 days, 10th‐90th percentile: 8‐24 days) (*P* < .001).

**Table 3:** Baseline characteristics before and after propensity score matching stage II to IV patients

	All patients (n = 26 095)					Propensity‐matched patients (n = 7476)				
	ODG (n = 22 291)	%	LDG (n = 3804)	%	Standardized difference	ODG (n = 3738)	%	LDG (n = 3738)	%	Standardized difference
Age (y)										
Median (IQR)	73 (65‐80)		71 (62‐78)		−0.19	70 (63‐78)		71 (62‐78)		0.01
<65	5436	24.4	1235	32.5	0.19	1219	32.6	1217	32.6	0.01
65‐75	7207	32.3	1197	31.5		1164	31.1	1174	31.4	
75<	9648	43.3	1372	36.1		1355	36.2	1347	36.0	
Gender										
Male	15 031	67.4	2491	65.5	0.04	2444	65.4	2450	65.5	0.00
Female	7260	32.6	1313	34.5		1294	34.6	1288	34.5	
ASA‐PS										
1,2	19 174	86.0	3449	90.7	−0.15	3395	90.8	3395	90.8	0.00
3,4,5	3117	14.0	355	9.3		343	9.2	343	9.2	
BMI (kg/m^2^)										
Mean (SD)	21.7 (3.4)		22.2 (3.4)		0.15	22.2 (3.3)		22.2 (3.4)		0.00
Weight loss 10%<	2337	10.5	166	4.4	−0.24	144	3.9	159	4.3	0.02
Smoking	4610	20.7	758	19.9	−0.02	670	17.9	744	19.9	0.05
Habitual alcohol intake	5157	23.1	1023	26.9	0.09	994	26.6	1010	27.0	0.01
Comorbidities										
Diabetes mellitus	699	3.1	108	2.8	−0.02	90	2.4	107	2.9	0.03
Respiratory	646	2.9	69	1.8	−0.07	55	1.5	65	1.7	0.02
COPD	987	4.4	142	3.7	−0.04	115	3.1	138	3.7	0.03
Hypertension	8098	36.3	1393	36.6	0.01	1344	36.0	1371	36.7	0.02
Ischemic heart disease	350	1.6	64	1.7	0.01	68	1.8	64	1.7	−0.01
Dialysis	160	0.7	17	0.4	−0.04	14	0.4	16	0.4	0.01
Cerebrovascular event	574	2.6	66	1.7	−0.06	60	1.6	64	1.7	0.01
Use of steroid	139	0.6	37	1.0	0.04	33	0.9	34	0.9	0.00
Bleeding disorder	957	4.3	127	3.3	−0.05	99	2.6	126	3.4	0.04
Surgical T										
T1a	27	0.1	17	0.4	0.49	13	0.3	14	0.4	0.01
T1b	148	0.7	64	1.7		62	1.7	62	1.7	
T2	2303	10.3	809	21.3		785	21.0	799	21.4	
T3	9347	41.9	1865	49.0		1864	49.9	1841	49.3	
T4a	9016	40.4	990	26.0		955	25.5	964	25.8	
T4b	1421	6.4	59	1.6		59	1.6	58	1.6	
Tx	29	0.1	0	0.0						
Surgical N										
N0	4501	20.2	1096	28.8	0.34	1099	29.4	1083	29.0	0.01
N1	5609	25.2	1202	31.6		1191	31.9	1188	31.8	
N2	5922	26.6	889	23.4		866	23.2	879	23.5	
N3a	4163	18.7	439	11.5		423	11.3	432	11.6	
N3b	1816	8.1	161	4.2		159	4.3	156	4.2	
NX	280	1.3	17	0.4						
Metastasis										
M0	18 915	84.9	3585	94.2	0.34	3544	94.8	3538	94.6	0.01
M1	3376	15.1	219	5.8		194	5.2	200	5.4	
Surgical stage										
IIA	4358	19.6	1363	35.8	0.53	1370	36.7	1347	36.0	0.03
IIB	4380	19.6	984	25.9		950	25.4	971	26.0	
IIIA	3517	15.8	553	14.5		569	15.2	545	14.6	
IIIB	3657	16.4	428	11.3		411	11.0	423	11.3	
IIIC	3003	13.5	257	6.8		244	6.5	252	6.7	
IV	3376	15.1	219	5.8		194	5.2	200	5.4	
Preoperative chemotherapy	1091	4.9	132	3.5	−0.07	105	2.8	129	3.5	0.04
Cholecystectomy	3745	16.8	307	8.1	−0.27	356	9.5	303	8.1	−0.05
Emergency surgery	351	1.6	17	0.4	−0.11	12	0.3	16	0.4	0.02

ASA‐PS, American Society of Anesthesiologists physical status; BMI, body mass index; COPD, chronic obstructive pulmonary disease; IQR, interquartile range; LDG, laparoscopic distal gastrectomy; ODG, open distal gastrectomy; SD, standard deviation.

Incidences of superficial SSI, deep SSI, and wound dehiscence were significantly higher in ODG compared to LDG as expected. The frequency of superficial SSI was 266 (1.8%) for ODG and 149 (1%) for LDG (*P* < .001). Deep SSI occurred in 89 (0.6%) of the ODG patients and in 55 (0.4%) of the LDG patients (*P* < .01). Moreover, the incidence of wound dehiscence in ODG was 59 (0.4%), whereas it was 27 (0.2%) in LDG (*P* < .001). Very interestingly, the incidence of pancreatic fistula was significantly higher in LDG (145: 1%) compared to ODG (116: 0.8%) (*P* = .01). There was no significant difference in the incidence of anastomotic leakage between ODG (1.8%) and LDG (1.9%).

### 3.3 Mortality and complications in stage II‐IV advanced GC patients

Surgical outcomes in stage II‐IV propensity‐matched patients (n = 7476) are shown in Table 4. We found no significant differences in mortality within 30 days and in‐hospital mortality between ODG and LDG. In addition, interestingly, there were also no statistically significant differences in other complications or in the incidences of reoperation and readmission after surgery. As shown for stage I patients, operating time in LDG (mean: 304.8 minutes, median: 296 minutes) was longer compared to ODG (mean: 230.4 minutes, median: 222 minutes) (*P* < .001), whereas blood loss was significantly higher in ODG (mean: 317.5 mL, median: 240 mL) compared to LDG (mean: 131.5 mL, median: 50 mL) (*P* < .001). Postoperative stay was significantly longer in ODG (median: 15 days, 10th‐90th percentile: 10‐35 days) compared to LDG (median: 13 days, 10th‐90th percentile: 8‐29 days) (*P* < .001).

**Table 4:** Surgical outcomes in stage II to IV patients

	ODG (n = 3738)	(%)	LDG (n = 3738)	(%)	*P*‐value
Operating time (min)					
Median [percentail 10‐90]	222 [139‐330]		296 [195‐427]		<.001
Blood loss (mL)					
Median [percentail 10‐90]	240 [57‐635]		50 [1‐308]		<.001
Mortality					
Within 30 days	11	0.3%	22	0.6%	.08
In‐hospital	33	0.9%	37	1%	.72
Readmission within 30 days	78	2.1%	93	2.5%	.28
Reoperation	106	2.8%	113	3%	.68
Complications					
Superficial SSI	70	1.9%	62	1.7%	.54
Deep SSI	21	0.6%	18	0.5%	.75
Intra‐abdominal abscess	109	2.9%	97	2.6%	.43
Leakage	82	2.2%	81	2.2%	1.00
Pancreatic fistula (grade B,C)	53	1.4%	55	1.5%	.92
Wound dehiscence	18	0.5%	10	0.3%	.18
Pneumonia	74	2%	55	1.5%	.11
Pulmonary embolism	8	0.2%	2	0.1%	.11
Sepsis	16	0.4%	17	0.5%	1.00
Length of postoperative stay					
Median [percentail 10‐90]	15 [10‐35]		13 [8‐29]		<.001

*P*‐values derived from Wilcoxon rank‐sum test for continuous variables and Fisher's exact test for binary variables.

LDG, laparoscopic distal gastrectomy; ODG, open distal gastrectomy; SSI, surgical site infection.

## 4 DISCUSSION

We made three major findings in the present study. First, during the study period, LDG was commonly carried out in patients with stage I GC, whereas open surgery was more common in more advanced cases; indeed, 85% or more of the stage II‐IV GC patients underwent ODG. Second, younger patients or those with lower ASA‐PS tended to be selected for a laparoscopic approach. Finally, after adjusting for confounding factors, neither the incidence of mortality nor that of morbidity was significantly higher for laparoscopic surgery than for the conventional open approach.

This was the first and largest‐scale survey to focus on the spread of laparoscopic surgery for patients with GC in Japan, so these findings should prove valuable and useful for surgeons in their daily practice. The current penetration of laparoscopic surgery has been limited to stage I GC, with a certain degree of safety ensured from the perspectives of mortality and morbidity. Although the conventional open approach was more common for stage II‐IV cancer during the study period, the proportion of patients undergoing laparoscopic surgery will likely increase rapidly in the near future under the revised Japanese treatment guidelines established in May 2014^3^ because the laparoscopic approach has been certified as a standard procedure for clinical stage I cancer. However, the oncological safety of laparoscopic surgery remains to be confirmed by phase III randomized trials; therefore, we will focus on the long‐term outcomes of on‐going clinical trials.^13^, ^14^ Indeed, some reliable observational studies have reported the non‐inferiority of the oncological outcomes of LDG compared with ODG.^15^, ^16^, ^17^, ^18^ We believe that this shift toward laparoscopic surgery will persist for some time, given the benefits associated with less‐invasive surgery. Given the situation described above, the present analysis was planned to confirm the safety of LDG in general practice in stage IA (T1N0) or IB (T1N1, T2N0) cancer, as defined in JCOG0703^19^ and JCOG0912,^13^ and to explore the feasibility of LDG for stage II‐IV cancer by a complete enumeration survey using the Japanese NCD registration system.

Apart from the TNM stage, there were some clinical factors associated with a surgeon's decision to select open or laparoscopic surgery for a given patient. In the present study, younger patients and those with better ASA‐PS tended to be treated by the laparoscopic approach, whereas a greater proportion of the patients with severe comorbidities received open surgery rather than laparoscopy, including those with diabetes mellitus, respiratory disease, hemodialysis, a history of cardiovascular events, and coagulation disorder. These trends were considered to reflect Japanese surgical practice.

Given these differences in patients’ characteristics, a number of confounding factors should be adjusted for when comparing surgical outcomes between laparoscopic and open surgery. After adjusting for confounding factors by precise propensity score matching, we ultimately noted no significant difference in the mortality of patients with stage I versus those with stage II‐IV cancer. Regarding morbidities, the incidences of SSI and pneumonia were common in ODG. Laparotomy with a long wound was likely to cause a SSI, which is consistent with the results of previous reports.^15^, ^20^, ^21^ In addition, difficulty of expectoration and rehabilitation after open surgery might be associated with postoperative pneumonia, which tended to be more frequent in the ODG group than in the LDG group. However, the greatest difficulty associated with LDG is the lack of tactile sensation experienced by the surgeon when manipulating the forceps. Although utmost caution should be paid to prevent organ injury as a result of the inappropriate use of forceps in LDG, the high incidence of pancreatic fistulas in LDG may be because of the assistant applying greater force than is actually required to displace the pancreas to expand the operative field and to the surgeon inflicting thermal injury on the pancreas by using energy devices.^22^ In addition, very interestingly, the rate of grade B or C pancreatic fistula was significantly higher in LDG (1.0%) than in ODG (0.8%) in stage I patients, whereas it was not markedly different between the two approaches in patients with stage II‐IV locally advanced cancer (ODG: 1.4%, LDG 1.5%). These are novel findings that have not been shown by other clinical research. The precise reason for the difference in outcomes between the different approaches in stage I cancer is unclear; however, pancreas injury during suprapancreatic lymph node dissection (mostly D1+ dissection) or compression of the pancreas by energy devices or forceps may be more frequent with LDG than with ODG. In stage II‐IV GC, more aggressive dissection at the D2 level in ODG increased the rate of pancreatic fistula, thereby reducing the statistical difference compared to LDG.

Significant differences in the incidence of morbidities were evident between the two procedures in this study, but the point estimation was small from the clinicians’ perspective. We should therefore be careful when interpreting this small *P‐*value in analyses using such a large‐scale dataset. Furthermore, we found that the length of hospitalization after surgery was shortened with LDG from 2.4 to 2.8 in‐hospital days compared with ODG. Taken together, the present findings suggest that the surgical safety and low invasiveness of LDG were mostly proven as already shown by considerable established evidence. However, as seen in Table 4, the merit of the laparoscopic approach in stages II to IV gastric cancer could not be shown. Expected lower invasiveness of LDG was not shown by the present analysis and, moreover, the cost of using LDG devices can be more expensive than that of ODG even if the hospital stay is short.

This analysis did not overcome all the uncertainties associated with the details of the surgical procedures, such as the degree of lymphadenectomy, methods or technique of reconstruction, or types of energy devices used. In addition, the NCD system did not include any variables related to nutrition, quality of life, or oncological outcomes. We should therefore pay careful attention to the results of ongoing clinical trials. Furthermore, we need to consider whether our results can be safely extrapolated to patients with GC worldwide. Also, the generalizability of our results to the population outside the PS matched cohorts (ie those with dominant propensity for either LDG or ODG) is unwarranted. There might be disadvantageous conditions related to the incidence of complications in Western countries, such as patients with a higher body mass index and the greater proportion of patients who present at an advanced stage.^23^, ^24^, ^25^ The surgical outcomes of the present survey have been gradually established and refined since 1991, when Kitano et al^2^ first reported LDG in patients with GC in Japan. Therefore, surgeons should continue to carefully consider the appropriate indications for laparoscopic surgery for GC.

In conclusion, we confirmed the surgical safety of LDG, which has similar incidences of mortality and morbidity to ODG, using the NCD registry system. In this first and complete enumeration survey from a Japanese national database, we confirmed that LDG is being conducted safely in Japan in stage I GC patients in general practice, as suggested by the Japanese guidelines for GC treatment. LDG may represent a new therapeutic option for patients with stage II‐IV disease as well as stage I GC patients.

## DISCLOSURE

Ethical statement: All procedures followed were in accordance with the ethical standards of the responsible committee on human experimentation (institutional and national) and in compliance with the Helsinki Declaration of 1964 and later versions. Both hospitals disclose information to the patients. Participating patients were excluded only when they specified that they were unwilling to participate.

Funding: This work was supported by grants for research promotion committee of the Japanese Gastric Cancer Association.

Conflicts of Interest: HK and HM are affiliated to the Department of Healthcare Quality Assessment at the University of Tokyo which is a social collaboration department supported by National Clinical Database and Johnson & Johnson K.K.; the department was formerly supported by endowments from Johnson & Johnson K.K., Nipro Corporation, Teijin Pharma Ltd., Kaketsuken K.K., St. Jude Medical Japan Co. Ltd., Novartis Pharma K.K., Taiho Pharmaceutical Co. Ltd., W. L. Gore Associates, Co. Ltd., Olympus Corporation, and Chugai Pharmaceutical Co. Other authors declare no conflicts of interest for this article.
